# Polylysine Enriched Matrices: A Promising Approach for Vascular Grafts

**DOI:** 10.3389/fbioe.2020.00281

**Published:** 2020-04-03

**Authors:** Luca Fusaro, Marta Calvo Catoira, Martina Ramella, Federico Sacco Botto, Maria Talmon, Luigia Grazia Fresu, Araida Hidalgo-Bastida, Francesca Boccafoschi

**Affiliations:** ^1^Department of Health Sciences, University of Eastern Piedmont Amedeo Avogadro, Novara, Italy; ^2^Tissuegraft srl, Novara, Italy; ^3^Center for Translational Research on Autoimmune and Allergic Diseases - CAAD, University of Eastern Piedmont Amedeo Avogadro, Novara, Italy; ^4^Physiology and Experimental Surgery, Department of Translational Medicine, University of Eastern Piedmont Amedeo Avogadro, Novara, Italy; ^5^Centre for Bioscience, Manchester Metropolitan University, Manchester, United Kingdom; ^6^Centre for Advanced Materials and Surface Engineering, Manchester Metropolitan University, Manchester, United Kingdom; ^7^Centre for Musculoskeletal Science and Sports Medicine, Manchester Metropolitan University, Manchester, United Kingdom

**Keywords:** decellularized vessels, vascular substitutes, polylysine, surface grafting, matrix degradation

## Abstract

Cardiovascular diseases represent the leading cause of death in developed countries. Modern surgical methods show poor efficiency in the substitution of small-diameter arteries (<6 mm). Due to the difference in mechanical properties between the native artery and the substitute, the behavior of the vessel wall is a major cause of inefficient substitutions. The use of decellularized scaffolds has shown optimal prospects in different applications for regenerative medicine. The purpose of this work was to obtain polylysine-enriched vascular substitutes, derived from decellularized porcine femoral and carotid arteries. Polylysine acts as a matrix cross-linker, increasing the mechanical resistance of the scaffold with respect to decellularized vessels, without altering the native biocompatibility and hemocompatibility properties. The biological characterization showed an excellent biocompatibility, while mechanical tests displayed that the Young’s modulus of the polylysine-enriched matrix was comparable to native vessel. Burst pressure test demonstrated strengthening of the polylysine-enriched matrix, which can resist to higher pressures with respect to native vessel. Mechanical analyses also show that polylysine-enriched vessels presented minimal degradation compared to native. Concerning hemocompatibility, the performed analyses show that polylysine-enriched matrices increase coagulation time, with respect to commercial Dacron vascular substitutes. Based on these findings, polylysine-enriched decellularized vessels resulted in a promising approach for vascular substitution.

## Introduction

Main cause of death worldwide is represented by cardiovascular diseases (Zoghbi et al., 2014), with a predicted annual incidence of cardiovascular disease-related mortalities expected to increase to 23.3 million worldwide by 2030 (Mathers and Loncar, 2006). These diseases are characterized by partial or complete occlusion of blood vessels lumen, leading to reduced blood flow and subsequent tissue damage and necrosis (Pashneh-Tala et al., 2015). The most common cardiovascular diseases are coronary heart diseases, cerebrovascular diseases, peripheral artery diseases, and deep vein thrombosis. When blood flow is significantly altered, leading to potential cardiovascular risks for patients, surgical intervention is required with the substitution of damaged vessels (Abdulhannan et al., 2012). In order to replace or bypass a damaged or occluded vessel, vascular grafts were introduced (Pashneh-Tala et al., 2015). Currently, the gold standard for vascular grafting are autologous arteries or veins, because of their natural biocompatibility, their non-thrombogenicity and their adequate mechanical characteristics (Farkouh et al., 2012). While arteries, such as the internal thoracic artery or radial artery, ensure a superior patency (Athanasiou et al., 2011; Masden et al., 2012), the saphenous vein is the most used autograft vessel (Harskamp et al., 2013). The use of saphenous veins is preferred because of the limited availability of adequately healthy arteries and the possible complications associated with arteries’ removal. On the other hand, autologous saphenous veins also show some drawbacks, such as their limited availability, due to the fact that the vessel could show poor quality, and its harvest could lead to damage or infection in the surgery site (Klinkert et al., 2004; Conte, 2013). Also, differences with the native vessel in compliance and elasticity often entail substitute’s occlusion, caused by atherosclerosis and cellular infiltration (Harskamp et al., 2013).

Another option for vascular surgery is the use of synthetic vascular grafts. Materials used for these grafts are mainly polymers, such as polyethylene terephthalate (PET and Dacron) and expanded polytetrafluoroethylene (ePTFE and Gore-Tex). Synthetic grafts used in large-caliber arteries (>8 mm), have demonstrated good results, such as in aortoiliac substitutes, where patency is shown to be around 90% (Faries et al., 2000) and in medium-caliber arteries (6–8 mm), such as carotid or common femoral artery replacements (Burger et al., 2000). Nevertheless, in small-caliber vessels (<6 mm) the use of synthetic grafts is limited due to poor success rates, thus in these cases autologous vessels are surely preferred. Small diameter grafts experience low and turbulent blood flow (Kannan et al., 2005), due to compliance differences between the synthetic substitute and native vessel at the graft site (Seifalian et al., 2003). Synthetic graft poor mechanical properties, added with no endothelial layer formation and poor hemocompatibility properties, lead to thrombus formation and luminal occlusion due to intimal hyperplasia, which explain poor patency rates of small-caliber synthetic grafts (Lemson et al., 2000; Kannan et al., 2005). Moreover, reduced circulation due to narrowing of peripheral grafts adds further challenges in maintaining adequate circulatory conditions (Couto et al., 2015; Amuzescu et al., 2016).

Due to the increasing demand for vessel substitution in severe arterial disease, and the fact that, for the reasons mentioned above, often the autologous graft is not possible, a biocompatible small-caliber vascular graft is required for surgical graft. Development of small-caliber vascular grafts must take into account several properties. The graft must be resilient and feature mechanical strength comparable to native vessels, with a burst pressure of ≥2.25 Bar (Seifu et al., 2013) to prevent aneurysm formation, and possessing comparable compliance with respect to native vessels, in order to avert intimal hyperplasia (Xue and Greisler, 2003). Also, luminal surfaces must allow the formation of a new endothelial layer, that will mediate vascular hemostasis (Seifalian et al., 2003). When established, the endothelium not only guarantees a perfect hemocompatibility, but also retains vessel integrity, directing the adjacent cell behavior via several signaling mechanisms. Indeed, in damaged endothelium the signaling loss is the main factor that leads to smooth muscle cell hyperproliferation, resulting in intimal hyperplasia and vessel occlusion, finally leading to graft failure (Jeschke et al., 1999; Melchiorri et al., 2013).

The use of decellularized biological matrices in regenerative medicine exploits the structure and mechanical properties of natural extracellular matrix (ECM) without yielding immunological reactions due to its biological origin. Decellularization process implies the removal of cellular residues from the tissue that could trigger antigenic reactions. Decellularization can be conducted with biological and/or chemical agents, such as enzymes, chelating agents, combinations of acids and bases, combinations of hypotonic and hypertonic solutions, detergents, solvents, in combination with physical agents such as agitation, pressure, and abrasion (Boccafoschi et al., 2017; Lin et al., 2018).

Preservation of the ECM also allows to preserve tissues’ mechanical properties (Conklin et al., 2002; Cho et al., 2005). Several clinical products obtained from human or animal decellularized tissues are commercially available for various applications, such as dermal replacement, soft tissue regeneration, as well as ophthalmic, orthopedic, and dentistry uses (Crapo et al., 2011).

Differently from synthetic grafts, decellularized matrices supply the appropriate environment for allowing cell repopulation, growth and differentiation. On the other hand, decellularized matrices present some altered mechanical properties, in terms of weakening of the vessel wall (Williams et al., 2009). This feature may eventually lead to implant failure, due to aneurysm formation and disturbances in the blood flow. One of the aims of regenerative medicine is to perform decellularization techniques that yield vascular grafts with mechanical characteristics of native vessels and positive immune properties of autologous vessels (Moroni and Mirabella, 2014).

Taking these considerations into account, the aim of the present study is to obtain a decellularized matrix, derived from femoral and carotid porcine arteries, and enrich it with polylysine, in order to strengthen the vessel wall while maintaining adequate biocompatibility of the scaffold.

## Materials and Methods

### Decellularization Process

Porcine femoral and carotid arteries, provided by Life and Device S.r.l. (Turin, Italy), have been decellularized with a three-step protocol. In summary, the vessels have been treated with a 1 M NaCl, 8 mM CHAPS and 25 mM EDTA solution for 1 h at 37°C under stirring. After prolonged PBS rinsing, the matrices have been incubated with a second solution of 1 M NaCl, 1.8 mM SDS and 25 mM EDTA for 1 h at 37°C under stirring. Finally, in order to remove the residual DNA content, samples have been treated with a solution of 6.4 μM Deoxyribonuclease I from bovine pancreas, 0.1 M MgCl_2_, and 0.9 M NaCl, and stirred for 16 h at room temperature (all reagents Sigma-Aldrich, United States). Decellularized vessels have been stored at −20°C before use.

### Functionalization of the Decellularized Biologic Tissue (sueGraft^®^ Process)

Decellularized matrices have been enriched with 1 mg/ml polylysine (Sigma-Aldrich, United States). The process (sueGraft^®^) used was optimized by TissueGraft S.r.l., all details are owned by the company.

To evaluate the efficiency of the enriching process, three different matrices were compared:

(1) Native vessel.
(2) Decellularized matrix.
(3) Polylysine grafting: decellularized matrix subjected to the enrichment process.

### Evaluation of the Decellularization Procedure

In order to verify the efficiency of the decellularization process, several analyses were performed (Wolf et al., 2012), including histology to evaluate the absence of cellular material using 4′, 6′-diamidino-2-phenylindole dihydrochloride (DAPI) and hematoxylin and eosin (H&E) staining. For the nuclear staining analysis (DAPI), samples were fixed in a 4% formaldehyde solution, then rinsed in PBS and soaked in DAPI solution (300 nM for 2 min). After an additional PBS rinsing, samples were observed under a fluorescence microscope.

For H&E staining, samples were fixed in 4% formaldehyde solution, dehydrated and paraffin embedded. Samples were then cut into sections of 5 μm, rehydrated and soaked in hematoxylin for 15 min. Following this, eosin solution (0.05% eosin in distilled water and acetic acid) was applied for 1 min. Finally, samples were dehydrated and observed with an optic microscope (Leica DM2500, Leica, Germany). Samples images have been acquired through a Leica DFC7000 T camera (Leica, Germany) and analyzed with Leica Application Suite X software (Leica, Germany).

In addition, the amount of DNA in each sample was also quantified through a DNA quantification assay. Native and decellularized vessel slices (5 mm × 5 mm) were lyophilized and digested for 16 h at 55°C in a buffer solution, containing 100 μg/ml proteinase K, 75 mM NaCl, 25 mM EDTA, 1% SDS, pH = 8. Samples were incubated with a 6 M NaCl solution for 30 min and centrifuged at 15,000 rpm; followed by 1 ml of isopropanol. In order to precipitate the DNA after centrifugation, the supernatant was removed, and 70% ethanol added. After evaporation of ethanol, 20 μl of ultrapure water was added and the amount of DNA measured with full-spectrum UV-visible measurements (NanoDrop 2000, Thermo Fisher Scientific, United States).

Finally, agarose gel electrophoresis was used to visualize the DNA fragmentation. Briefly, 50 ng of DNA from native and decellularized samples were mixed with EZ-Vision loading buffer (VWR, Italy), and loaded on a 1% agarose gel. After the electrophoresis, DNA was visualized with ChemiDoc Imaging System (Bio-Rad, Italy).

### Enrichment Process Assessment and Morphological Analysis

#### X-Ray Photoelectron Spectroscopy (XPS)

X-ray Photoelectron Spectroscopy (XPS) analysis was conducted with a PerkinElmer PHI 5600 ESCA system. The instrument is supplied with a dual Al (monochromatic) and Mg anode operating at 10 kV and 200 W. The diameter of the analyzed spot was about 500 μm, the sampling depth was 5–8 nm, and the base pressure 10^–8^ Pa. The angle between the electron analyzer and the sample surface was 45 degrees.

After preliminary evaluation using the monochromated X-ray source, spectra were acquired using the non-monochromated Mg anode, given side-effects due to charging were much lower. Analysis was conducted acquiring wide range survey spectra (0–1,000 eV binding energy) and detailed high-resolution peaks of relevant elements.

Quantification of elements was completed using the software and sensitivity factors furnished by the manufacturer.

#### Attenuated Total Reflectance Infra-Red Spectroscopy (ATR-IR)

ATR-IR spectra were obtained using a Nicolet iS10 ATR-IR spectrometer with a diamond crystal (Thermo Fisher Scientific, United States). Samples were placed on the crystal and held steady by the specific crimping tool. The experimental set up involved acquisition of 32 scans in the range 500–4000 cm^–1^, both of sample and background, at a resolution of 4 cm^–1^ with the sampling depth is approximately 1.6 μm. Beside decellularized and polylysine-enriched samples, the ATR-IR spectrum of polylysine (Sigma-Aldrich, United States) was acquired as a reference.

#### Confocal Microscopy

The microscopy visualization of the structures was completed using a Leica TCS SP8 DLS (Digital LightSheet, CLF, Harwell, United Kingdom) system with image acquisition technical information as shown in Table 1. The focus of this tool was the observation of the scaffold collagen structures under static conditions. Samples were not stained but fixed *in situ* with agar to immobilize the sample during the test.

**TABLE 1:** Technical data of Figure 3A images.

	**A**	**B**	**C**	**D**
Field of view	1471.52 μm × 1471.52 μm	1471.52 μm × 1471.52 μm	735.76 μm × 735.76 μm	735.76 μm × 735.76 μm
Number of spaced steps	127 steps spaced of 6 μm	212 steps spaced of 6 μm	394 steps spaced of 1.5 μm,	342 steps spaced of 1.5 μm
Illumination laser	405 nm	405 nm	405 nm	405 nm
Laser power	25 μW	25 μW	25 μW	25 μW
Exposure time per plane	600 ms	600 ms	300 ms	600 ms
Bandpass detection filter	504–545 nm	504–545 nm	504–545 nm	504–545 nm
Detection objective	HC PL FLUOTAR 5×/0.15 IMM	HC PL FLUOTAR 5×/0.15 IMM	HC APO L 10×/0.30 WATER	HC APO L 10×/0.30 WATER
Illumination objective for light sheet creation	1.6×/0.05 DRY	1.6×/0.05 DRY	HC PL FLUOTAR 2.5×/0.07 DRY	HC PL FLUOTAR 2.5×/0.07 DRY

#### Micro Computed Tomography (Micro-CT)

A micro-CT system (High Flux Nikon XTEK Bay), Manchester X-ray Imaging Facility (MXIF) was used to non-destructively image and visualize the three-dimensional micro-morphology as previously described (Metscher, 2009; Disney et al., 2017). Staining method used was 1% (w/v) phosphotungstic acid, H_3_PW_12_O_40_ (PTA) in water for 40 h followed by washing in 70% ethanol and dried for 48 h at room temperature.

Samples were mounted on a stage within the imaging system and subsequently scanned at a voltage of 100 kV and a current of 50–100 mA. Specimens were scanned at a 2 μm resolution with an integration time of 1,000–1400 ms to produce 3D images. Raw 2D images were thresholded to remove background values (threshold value = 60) and further analyzed using Avizo software (Thermo Fisher Scientific, United States) (Walton et al., 2015).

### Mechanical Test

Mechanical properties of native and decellularized enriched matrices were tested. The Young’s modulus and tensile strength of the different samples was measured, briefly, Young’s moduli were quantified from the stress-strain curve obtained with the Instron 5564 (Instron Corporation, Norwood, MA, United States). System control and data analyses were performed using Instron^®^ Bluehill^TM^ 3 material-testing software (Instron Corporation, Norwood, MA, United States). Sample dimensions (length, width, and thickness) were measured in order to normalize the mechanical test parameters. Uniaxial tests were performed at room temperature and with a tension rate of 0.5 mm^∗^s^–1^.

Burst pressure was also measured. The test was performed using a hydraulic press, a high-precision digital pressure gauge and a PBS solution as a liquid. The sample was stressed by pushing the PBS until bursting, thus detecting the maximum operating pressure in laboratory conditions.

Mechanical properties were also evaluated after simulation of physiological conditions (dynamic condition test). Decellularized and polylysine-enriched vessels have been inserted in a circuit, composed of a peristaltic pump (Masterflex L/S, Cole-Parmer Instrument Company, United States), 10 mm diameter tubing (Cole-Parmer Instrument Company, United States), and a chamber containing the samples and filled with the PBS. Fluid speed was 15 ml^∗^min^–1^ and samples were tested for up to 30 days. Samples were weighed before and after the dynamic test in order to evaluate the samples’ weight loss after dynamic conditions were applied. Finally, both Instron and burst pressure tests, were performed after dynamic conditions.

### Cell Viability Assay

Cell viability of human umbilical vein endothelial cells EA.hy926 (ATCC^®^ CRL-2922^TM^), cultured on decellularized and enriched matrices, was assessed. Cells were cultured with Dulbecco’s Modified Eagle’s Medium (DMEM – Euroclone, Italy) enriched with 10% fetal bovine serum (FBS), 2 mM glutamine and 100 U^∗^mL^–1^ penicillin (Euroclone, Italy). Endothelial cells were cultured at 5^∗^10^4^ cells^∗^cm^–2^. In order to evaluate cell viability, after 1, 3, and 7 days of culture, a MTS assay (CellTiter 96^®^ AQueous Non-Radioactive Cell Proliferation Assay, Promega, Italy) was performed. Briefly, a 3-(4,5-dimethylthiazol-2-yl)-5-(3-carboxymethoxyphenyl)-2-(4-sulfophenyl)-2H-tetrazolium solution was added to each sample for 3 h. Absorbance was measured by UV-VIS spectrophotometry (Victor X4, PerkinElmer, United Kingdom), at a wavelength of 490 nm. Measures were proportional to cell viability.

In order to evaluate cell adhesion and proliferation, EA.hy926 cells were cultured on decellularized and polylysine enriched matrices for 3 and 7 days. Matrices were then observed through Hematoxylin-eosin staining, as described in Section “Evaluation of the Decellularization Procedure.”

Cell adhesion was evaluated after simulation of physiological conditions (dynamic condition test). EA.hy926 were cultured on the lumen side of decellularized and polylysine-enriched vessels for 24 h. Afterward, samples were inserted in a circuit, composed of a peristaltic pump (Masterflex L/S, Cole-Parmer Instrument Company, United States), 10 mm diameter tubing (Cole-Parmer Instrument Company, United States), and a chamber containing the vessels samples and filled with DMEM enriched with 10% FBS. Fluid speed was 15 ml^∗^min^–1^ and samples were tested for up to 3 days.

### Monocyte Activation

Monocyte viability and inflammatory response elicited by decellularized and polylysine-enriched matrices was evaluated. After 24 h, cell viability on samples was evaluated with MTT assay. Briefly, culture medium was replaced with a 5 mg/ml solution of Thiazolyl Blue Tetrazolium Blue (MTT, Sigma-Aldrich, Italy) in RPMI 1640 and incubated for 4 h at 37°C. Medium was removed and DMSO was added, in order to dissolve formazan salts. Absorbance was measured by UV-VIS spectrophotometry (Victor X4, PerkinElmer, United Kingdom), at a wavelength of 570 nm. Absorbance is proportional to cell viability.

To evaluate the effect of decellularized and enriched samples on monocytes activity and polarization, cells on samples and controls (M0, M1, and M2) were seeded with 2^∗^10^5^ cells^∗^cm^–2^. Differentiation of monocytes in control samples was obtained adding 10% FBS-enriched RPMI 1640 medium (Euroclone, Italy) with human (hr)GM-CSF (50 ng⋅mL^–1^, Sigma-Aldrich, Italy), to induce M1 macrophages polarization, and in 10% FBS-enriched medium containing hrM-CSF (50 ng⋅mL^–1^ Sigma-Aldrich, Italy) to induce M2 macrophages polarization. These conditions have been maintained for 7 days.

After 7 days, culture medium from each sample was collected and TNF-α release was evaluated through ELISA assay (RAB0476, Sigma Aldrich, Italy). Analysis was performed according to manufacturer’s instructions. Absorbance was measured by UV-VIS spectrophotometry (Victor X4, PerkinElmer, United Kingdom), at a wavelength of 450 nm. Measures were proportional to TNF-α concentration.

### Hemocompatibility

Thromboelastography (TEG) is a technique that measures several parameters relative to blood coagulation by evaluating the viscoelastic properties of blood from the beginning of coagulation to the clot rupture with fibrinolysis. Particularly important parameters are the coagulation formation, its progression, maximum strength, and stability, which provides important information on coagulation, fibrinolysis, and platelet functionality. Blood samples were collected in Vacutainer (BD, United States) containing sodium citrate and tested at 37°C. Blood was exposed to the control graft (Dacron^®^), the decellularized scaffold and the enriched scaffold for 30 min and subsequently analyzed using Thromboelastograph^®^ (TEG^®^ 5000 Thrombelastograph^®^ Hemostasis Analyzer System) and the Kaolin TEG standard protocol. Briefly, after 30 min 1 ml of blood was collected and placed in a vial containing kaolin. After few seconds, 340 μl of blood were sampled in the thrombelastograph with 20 μl of calcium chloride, in order to eliminate the anticoagulant effect of sodium citrate. Coagulation parameters were evaluated through thromboelastogram obtained at the end of the testing time.

## Results

### Decellularization Process Efficiency

**FIGURE 1:**
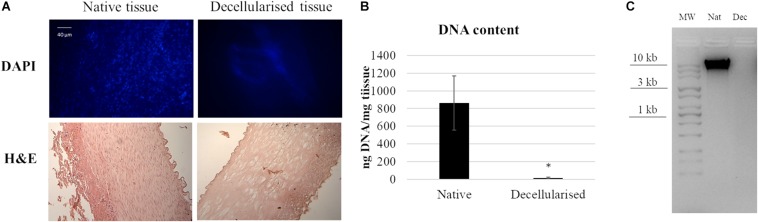
Decellularization of porcine artery showing **(A)** DAPI and HandE histology before and after decellularization process. **(B)** Quantitative determination of DNA in a native and decellularized matrix. Data are expressed as mean values ± SD (*n* = 3, *p ≤ 0.05). **(C)** Agarose gel electrophoresis of DNA derived from native and decellularized matrices. The image is representative of three different analyses.

In order to evaluate the efficiency of the decellularization protocol, DAPI staining was performed on native and decellularized tissue. Figure 1 shows the native tissue and the presence of cellular nuclei in native tissues while, after the decellularization treatment, however, no nuclei were present. H&E histology results confirmed the preservation of the extracellular matrix microstructure within the decellularized samples. No cellular nuclei could be detected in the decellularized tissue samples.

DNA quantification on native and decellularized tissues strengthened the previous results, indicating a successful DNA removal from the matrix. In contrast to native tissue, which contained 860 ± 300 ng DNA per mg wet weight (*n* = 3), decellularized tissues showed a significant decrease of DNA content to 15 ± 11 ng DNA per mg wet weight (*n* = 3). Agarose gel showed that no DNA strands longer than 100 bp were available on decellularized samples, while in native samples the DNA presence was verified.

### Enrichment Process Efficiency and Morphological Analysis

**FIGURE 2:**
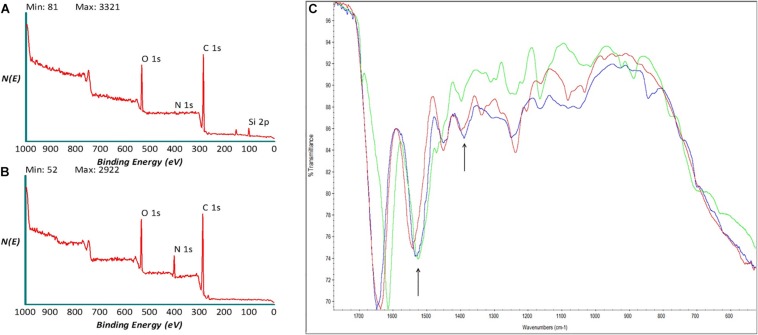
X-ray photoelectron spectroscopy (XPS) spectra of **(A)** decellularized and **(B)** polylysine enriched matrices. **(C)** AT-IR spectra of decellularized (red), polylysine enriched matrices (blue), and Polylysine as a control (green).

Samples were analyzed, as shown in Figures 2A,B, the survey spectra of decellularized and polylysine-enriched samples show differences, with the obtained surface composition reported in Table 2. Decellularized scaffolds showed peaks due to carbon, oxygen and silicon, and just a very weak signal from nitrogen. XPS tests just the outermost molecular layers of materials (the sampling depth is about 5–8 nm), and the obtained data likely reflects surface side-effects due to processing. Silicone is a common contaminant of surfaces and the peak of Si is a common finding in surface analysis of biological samples. There was also a lower than expected N/C and N/O ratio, which was probably due to processing aids (see also discussion of ATR-IR results) remaining on or combined with the sample surface. In contrast, the XPS spectrum of polylysine-enriched samples showed a strong peak due to nitrogen. Hence, the sample surface showed being enriched with a nitrogen-containing compound, clearly supporting the presence of polylysine. Further information was provided by ATR-IR analysis, as reported in the next section.

**TABLE 2:** Surface composition (atomic%) of tested samples (*n* = 3).

**Sample**	**O**	**N**	**C**	**Si**
Decellularized	17.1	1.4	68.4	13.1
Polylysine	16.7	11.3	72.0	–

ATR-IR analysis of decellularized (red), polylysine-enriched (blue), and reference polylysine (green) in the amide band region of the IR spectrum (Figure 2C). The sampling depth of ATR-IR, using a diamond crystal, is around 1.6 mm, that is this technique is less surface-sensitive than XPS. All samples show the typical spectral features due to amide bonds, as expected. Concerning differences between tested samples, the Amide II band at about 1,550 cm^–1^ shifts toward lower wavenumbers from decellularized to polylysine-enriched (arrow), centering close to the Amide II band of the polylysine reference. Also, around 1,400 cm^–1^ the shape of the band (arrow) changes from decellularized to polylysine. Taken together, XPS and ATR-IR results strongly suggest that successful enrichment with polylysine of the decellularized sample is obtained through this process.

**FIGURE 3:**
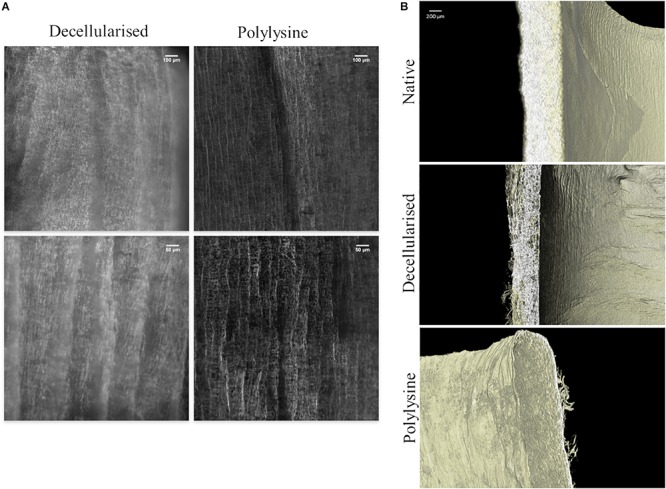
**(A)** Confocal microscopy of decellularized and polylysine-enriched vessels. **(B)** Micro-CT analysis of native vessel, decellularized and polylysine-enriched matrices.

In Figure 3A the structure of decellularized and polylysine-enriched matrices are shown through confocal microscopy. The polylysine-enriched sample shows a structure of fractures better defined than in the decellularized sample, and the roughness is narrower than the latter. In general, the enriched sample shows more vertical oriented fractures, while in decellularized the lines are not so straight composing soft curves. The images suggest that the enrichment treatment changes the micro and macro collagen structure, orientating and bridging collagen fibers, as can be seen in Figure 3B, which confirms the data obtained from confocal microscopy. Indeed, decellularized sample’s tissue seems thinner and hollower with respect to both native and polylysine enriched matrix. The thickness and the wall microfiber structure of polylysine enriched matrix seems adequately similar, suggesting that the fiber reorganizing after enrichment process leads to a structure comparable to native sample.

### Mechanical Test

#### Young’s Modulus, Tensile Strength and Burst Pressure Results: Static Conditions

**FIGURE 4:**
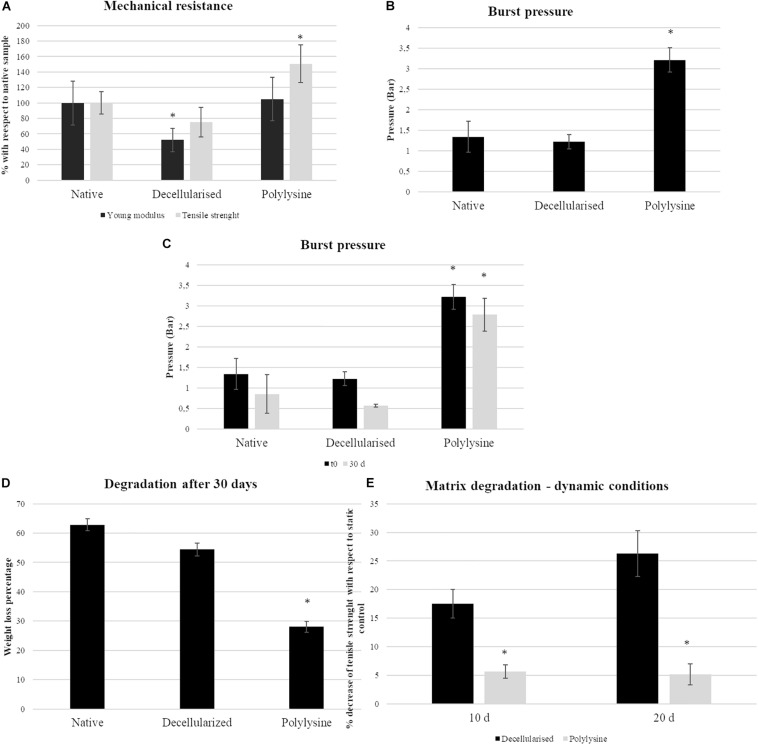
Mechanical testing results under static conditions: (**A**, left panel) Young’s modulus and tensile strength representation for native, decellularized and polylysine grafting. Data are expressed normalized with a native vessel. The data origin is the stress–strain curves. Data are expressed as mean values ± SD (*n* = 3, ^∗^*p* ≤ 0.05). **(B)** Burst pressure data for native, decellularized and polylysine grafting. Data are expressed as mean values ± SD (*n* = 3, ^∗^*p* ≤ 0.05). Mechanical testing results under dynamic conditions: **(C)** Burst pressure data for native, decellularized, and polylysine grafting for static and dynamic conditions. Data are expressed as mean values ± SD (*n* = 5, ^∗^*p* ≤ 0.05). **(D)** Degradation assays in terms of weight loss. Data are expressed as mean values ± SD (*n* = 5, ^∗^*p* ≤ 0.05). **(E)** Degradation assays in terms of tensile strength loss. Data are expressed as mean values ± SD (*n* = 3, ^∗^*p* ≤ 0.05).

To characterize the mechanical properties (Figures 4A,B) specimens were tested, using an Instron instrument, and strained until specimen failure.

The Young’s modulus is directly related to the compliance of the material. Decellularized samples present a significant Young’s modulus decrease when compared with native and polylysine sample values. On the other hand, the tensile strength of the polylysine enriched matrix is increased, compared to the native vessel, while the same parameter between the decellularized matrix is lower with respect to the other samples.

The figure shows that, with respect to the native control, elasticity is not affected. The Young’s modulus of the enriched matrix is shown to be not significantly different to the native vessel. The scaffold wall is strengthened after enrichment.

#### Young’s Modulus, Tensile Strength and Burst Pressure Results: Dynamic Conditions

Samples were tested for up to 30 days under dynamic conditions, with a PBS flow inside the vessel lumen. After performing dynamic tests, matrix degradation was measured. All the samples reveal a weight loss: native samples’ mass decreases of 60%, while decellularized samples roughly 55%. Nevertheless, the polylysine samples present only a 28% weight loss, with an evident matrix preservation compared to other samples (Figure 4C).

Burst pressure was measured before and after applying dynamic conditions. Native artery pressure is 1.3 bar, while with polylysine treatment, the value is considerably increased, rising to 3.2 bar.

After 30 days, the maximum pressure value supported by the samples decreases by 40% in the native sample and only a 13% in the sample treated with polylysine (Figure 4D).

The successful performances of polylysine enriched matrices are confirmed by the evaluation of matrix degradation in terms of tensile strength loss. Indeed, after 10 days polylysine enriched matrices lose only 5% of tensile strength while, decellularized matrix is 17% weaker than the static control. The differences are more evident after 20 days where decellularized matrices lose 26% of tensile strength, while treated decellularized vessels showed similar performances with respect to 10-day samples (Figure 4E).

### Cell Viability Assay: Static and Dynamic Conditions

**FIGURE 5:**
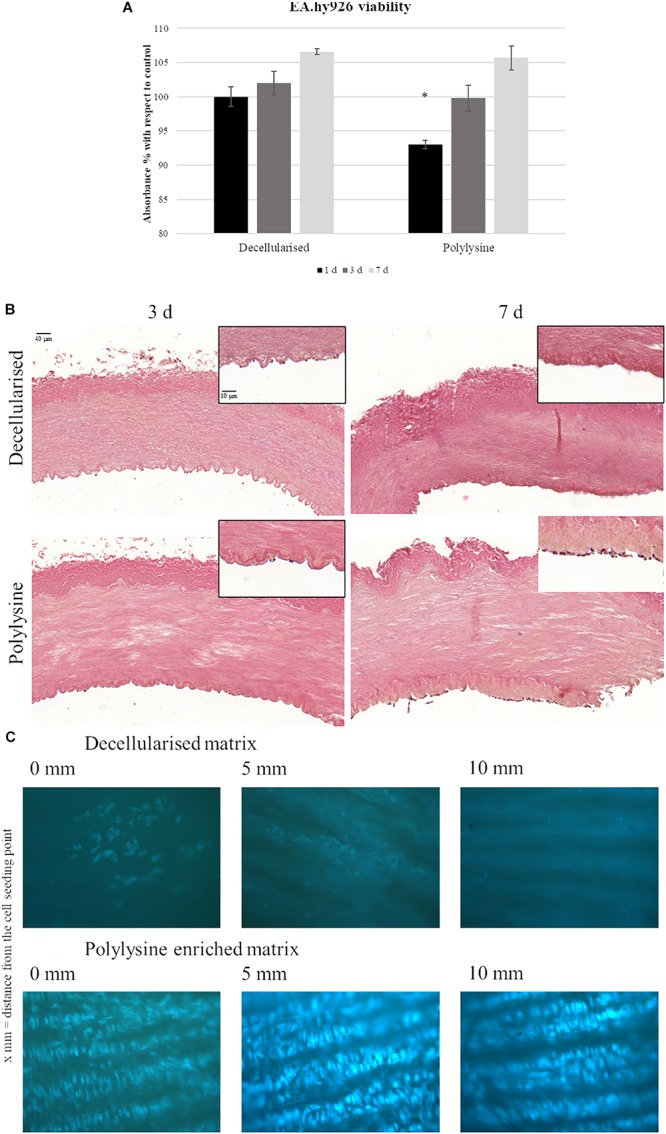
**(A)** Endothelial cells viability assays. MTS test was performed after 1, 3, and 7 days. Data are expressed as mean values ± SD (*n* = 3, ^∗^*p* ≤ 0.05). **(B)** Hematoxylin-Eosin staining of decellularized and polylysine enriched matrices with cells cultured for 3 and 7 days. **(C)** DAPI staining of decellularized and polylysine enriched matrices after dynamic conditions.

Endothelial cells were seeded in the vessels lumen and cell proliferation rate was measured after 1, 3, and 7 days. Figure 5A shows comparable cell proliferation rates for decellularized and polylysine-enriched samples after 7 days. Endothelial cells’ adhesion and proliferation were also evaluated, using hematoxylin-eosin and DAPI staining. Results show that after 3 days, cells adhered on both decellularized and polylysine-enriched samples, while after 7 days of culture cells formed an almost continuous monolayer only on polylysine-enriched surfaces (Figure 5B). Finally, cell adhesion was evaluated after 3 days of dynamic conditions (Figure 5C). DAPI staining shows almost no cell nuclei on decellularized samples, indicating that cells do not remain adherent to that matrices. On the other hand, polylysine-enriched samples show cell nuclei almost forming a continuous monolayer, suggesting that cell adhesion is optimized on enriched matrices.

### Monocyte Activation

**FIGURE 6:**
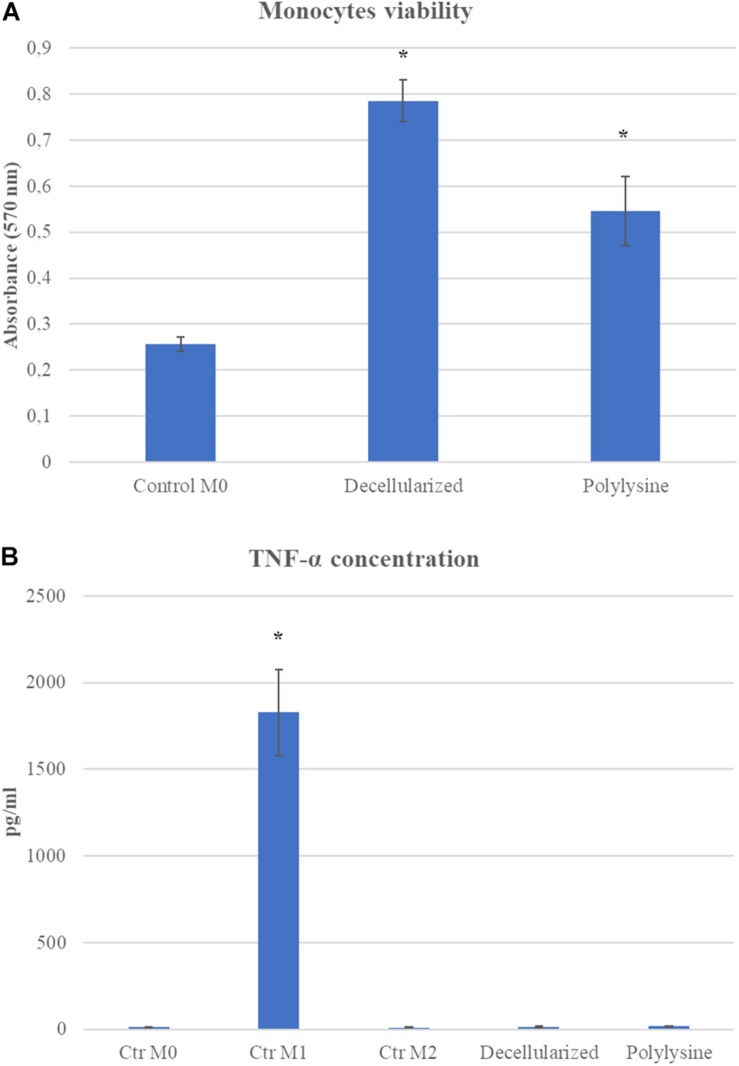
**(A)** Monocyte viability assay. MTT assay was performed after 24 h. **(B)** ELISA assay on TNF-α concentration after 7 days of culture. Data are expressed as mean values ± SD (*n* = 3, *p ≤ 0.05).

Monocyte viability on decellularized and enriched matrices was evaluated after 24 h after seeding. After 24 h, results show viable monocytes growth on control, decellularized and enriched matrices. Monocytes in contact with decellularized and enriched matrices showed an excellent viability. Thus, we proceeded analyzing monocytes polarization after 7 days of culture. Adequate media were used to induce macrophages polarization in M1 and M2, and used as control with respect to results obtained on macrophages growth on decellularized and enriched samples. As shown in Figure 6, M1 control released a significant amount of TNF-α, expressing an inflammatory phenotype, as expected. All the other conditions, both in M0 and M2 controls, as well as on decellularized and enriched matrices, TNF-a release is not significant, confirming the absence of inflammatory environment (Figure 6).

### Hemocompatibility Test

**FIGURE 7:**
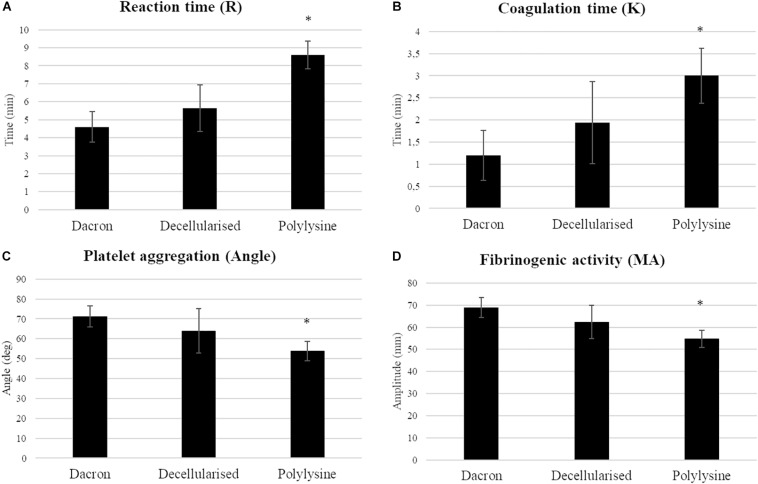
Haemocompatibility test. Coagulation parameters (**A:** reaction time; **B:** coagulation time; **C:** platelet aggregation; **D:** fibrinogenic activity) were evaluated on synthetic vascular substitutes (DACRON) and treated matrices. Data are expressed as mean values ± SD (*n* = 3 from different healthy donors, ^∗^*p* ≤ 0.05).

We evaluated the materials’ hemocompatibility using thromboelastography (TEG). Among the coagulation parameters, reaction time (*R*) indicates the time required to induce the clot formation. The matrices treated with polylysine have a significantly increased *R*-value with respect to Dacron and decellularized matrices. Fibrinogenic activity is rather decreased on enriched matrices, as a result the clot strength is lower than both matrices used as reference. The coagulation time parameter is directly correlated with the required time for the clot formation, in order to reach the maximum strength value before starting the dissolution process. This value increases in the samples treated with polylysine reflecting a benefit in terms of functionality. In addition, platelet aggregation confirms the lower rate in the clot formation process (Figure 7).

These results clearly indicate a favorable influence on coagulation parameters in the presence of polylysine enrichment in terms of hemocompatibility.

## Discussion

Although autologous arterial and venous grafts are the gold standard as bypass graft material, due to insufficient length or quality of native vessels for roughly 40% of patients requiring arterial bypass surgery autologous grafts are not available (Moroni and Mirabella, 2014).

Synthetic grafts, including Dacron and ePTFE, which efficiently work for large-diameter vascular grafts, have shown disappointing clinical results in small-diameter artery reconstruction, due to thrombotic complications when compared to venous bypasses (Klinkert et al., 2004). For these reasons, decellularized scaffolds were introduced. The ECM is an optimal substrate in terms of biological performances, because ECM is able to drive cell adhesion, proliferation, and differentiation (Barnes et al., 2011); in this way, unlike synthetic materials, decellularized substitutes are able to solve a complex problem as the repopulation with different cell types, as showed by Pellegata et al. (2013). On the other hand, decellularized matrices progressively lose the suitable mechanical properties needed for tissue replacement (Williams et al., 2009; Xiong et al., 2013). In the present study, we exploited the properties of the decellularized matrices, strengthened with polylysine enrichment, to prevent wall weakening (Gu et al., 2018). Polylysine is a polymer that has been widely used in tissue engineering, as a cell adhesion promoter, because of strong positive charge (Wang et al., 2018; Zhang et al., 2019) and as a biomaterial strengthener (Collin et al., 2004; Wang et al., 2016).

Evaluation of the effectiveness of decellularization process showed that no cell nuclei are present in decellularized matrices, and the DNA quantity is significantly lower than the native vessel, as well as also being lower than the gold standard defined by literature (Gilbert et al., 2009). However, it is unclear if the DNA left in decellularized matrices could elicit a biological response from the host (Keane et al., 2012). Several commercial products derived from decellularized matrices with positive clinical outcomes contain DNA fragments, for this reason the probability that residual DNA could induce a host response is low (Pellegata et al., 2013; Gu et al., 2018).

In order to predict the possible outcome of a vascular substitution, mechanical properties are a crucial parameter to evaluate. In particular, graft failure occurs when the substitute’s compliance is different from the native vessel (Xue and Greisler, 2003). Although Young modulus and failure strength of these substitutes are not significantly different from native vessels, the compliance was not taken into account as a key factor (Bergmeister et al., 2005; Ketchedjian et al., 2005; Mahara et al., 2015). On the contrary, Xue and Greisler (2003) showed that, in human patients graft failure occurs when the substitute’s compliance is different from the native vessel hyperplasia. For this reason, in the present study differences in compliance between polylysine-enriched substitute and the native vessel were evaluated. As known from literature, compliance is also related to wall stiffness (Baltgaile, 2012), which can be evaluated by Young’s modulus. Mechanical characterization of the matrices in static conditions showed that polylysine enriched matrix showed increased wall strength, while Young’s modulus is similar to native vessels. The assessment of mechanical properties shows that, given the similarities in stiffness between native vessel and polylysine enriched matrices, the compliance is comparable.

In order to assess the durability of the enrichment and the behavior of the substitute under dynamic conditions, degradation rates were also evaluated, as weight loss and tensile strength loss. In both cases, polylysine enriched vessels showed the best behavior, retaining matrix and mechanical properties, while all the other matrices considered lose their properties after 30 days. The importance of mechanical properties evaluation is underlined in Williams et al. (2009) who study the differences in stiffness between fresh and decellularized rabbit carotid arteries; also Mahara et al. (2015) focus on difference in stress strain curve between original tissue and peptide-enriched decellularized ostrich carotid artery graft; finally, López-Ruiz et al. (2017) evaluate tensile strength, maximum load and burst pressure of native, decellularized and coated porcine carotid arteries.

Treated matrix cytocompatibility showed to be adequate, compared to decellularized matrices, indicating a favorable environment for efficient repopulation by the host vascular cells. The adequacy of modified decellularized matrices was also demonstrated by Mahara et al. (2015) who indicate adequate cell repopulation of peptide-enriched decellularized grafts in porcine model; also Boerboom et al. (2002) confirmed good recellularization of decellularized carotid artery implant; finally Hilbert et al. (2004) further evidentiates successful repopulation of decellularized carotid goat graft. Moreover, treated matrices seem to favor recruitment of monocytes, while not inducing inflammatory phenotype expression. Further analyses will focus on macrophages polarization, in order to verify the potentiality of this matrices to induce a regenerative response more than an inflammatory response. In fact, this aspect is important because it is demonstrated that a positive immune system response contributes to the implant success (Hibino et al., 2015). Hemocompatibility tests displayed that polylysine enriched matrices develop blood clots slower and with less strength with respect to both decellularized matrices and Dacron, a synthetic polymer widely used in vascular substitution. Furthermore, coagulation assays indicate that polylysine enriched matrices could help to avoid thrombosis, another drawback of synthetic substitute (Fukunishi et al., 2016).

Given the positive results obtained by these *in vitro* experiments, these polylysine enriched substitutes are under preclinical evaluation using ovine models.

## Conclusion

Overall, our tests confirm the efficiency of both decellularization and enrichment methods, as well as the ability of polylysine enriched matrices to maintain adequate mechanical properties, while enhancing cell adhesion and proliferation. Thus, the enriched biological substitutes could represent an innovative approach for application in vascular tissue engineering.

## Data Availability Statement

All datasets generated for this study are included in the article/supplementary material.

## Author Contributions

LF, MC, MR, MT, LGF, and FB contributed to the design and implementation of the research, to the analysis of the results and to the writing of the manuscript. FSB contributed to the development of the experiments. AH-B contributed to the design of the research and the writing of the manuscript.

## Conflict of Interest

LF, MC, and MR were employed by company TissueGraft s.r.l. The remaining authors declare that the research was conducted in the absence of any commercial or financial relationships that could be construed as a potential conflict of interest.
